# VPAC: Variational projection for accurate clustering of single-cell transcriptomic data

**DOI:** 10.1186/s12859-019-2742-4

**Published:** 2019-05-01

**Authors:** Shengquan Chen, Kui Hua, Hongfei Cui, Rui Jiang

**Affiliations:** 1 MOE Key Laboratory of Bioinformatics; Bioinformatics Division, Beijing National Research Center for Information Science and Technology; Department of Automation, Tsinghua University, Beijing, 100084 China; 2 Institute for Artificial Intelligence and Department of Computer Science and Technology, Tsinghua University, Beijing, China

**Keywords:** Single-cell, scRNA-seq, Clustering, Variational projection, Dimensionality reduction, Multi-scale, Dropout, Cell subtypes

## Abstract

**Background:**

Single-cell RNA-sequencing (scRNA-seq) technologies have advanced rapidly in recent years and enabled the quantitative characterization at a microscopic resolution. With the exponential growth of the number of cells profiled in individual scRNA-seq experiments, the demand for identifying putative cell types from the data has become a great challenge that appeals for novel computational methods. Although a variety of algorithms have recently been proposed for single-cell clustering, such limitations as low accuracy, inferior robustness, and inadequate stability greatly impede the scope of applications of these methods.

**Results:**

We propose a novel model-based algorithm, named VPAC, for accurate clustering of single-cell transcriptomic data through variational projection, which assumes that single-cell samples follow a Gaussian mixture distribution in a latent space. Through comprehensive validation experiments, we demonstrate that VPAC can not only be applied to datasets of discrete counts and normalized continuous data, but also scale up well to various data dimensionality, different dataset size and different data sparsity. We further illustrate the ability of VPAC to detect genes with strong unique signatures of a specific cell type, which may shed light on the studies in system biology. We have released a user-friendly python package of VPAC in Github (https://github.com/ShengquanChen/VPAC). Users can directly import our VPAC class and conduct clustering without tedious installation of dependency packages.

**Conclusions:**

VPAC enables highly accurate clustering of single-cell transcriptomic data via a statistical model. We expect to see wide applications of our method to not only transcriptome studies for fully understanding the cell identity and functionality, but also the clustering of more general data.

## Background

Single-cell RNA-sequencing (scRNA-seq) has emerged as a revolutionary tool to reveal previously unknown heterogeneity and functional diversity at a microscopic resolution. Since the first protocols were published in 2009 [1], a massive expansion in method development has derived scRNA-seq technologies with distinct advantages and applicability [2]. For example, Smart-seq2 [3] and MARS-seq [4] is preferable when quantifying transcriptomes of fewer cells, while Drop-seq [5] is preferable when quantifying transcriptomes of large numbers of cells with low sequencing depth [6]. Advances in scRNA-seq technology have resulted in a wealth of studies aiming to reveal new cell types [7, 8], assess tissue composition [4, 9, 10], identify gene regulatory mechanisms [11, 12], investigate cell development or lineage processes [13–15], and many others. With the exponential growth of the number of cells profiled in individual scRNA-seq experiments, there is a demand for novel analysis methods for this new type of transcriptomic data, which has not only much greater scale of datasets than that of bulk experiments but also various challenges unique to the single-cell context [16].

A key advantage of scRNA-seq is that it can be used to identify putative cell types using unsupervised clustering, which is essential to fully understand the cell identity and functionality. A variety of algorithms have recently been proposed for single-cell clustering. For example, CellTree produces tree structures outlining the hierarchical relationship between single-cell samples using a novel statistical approach based on document analysis techniques [17]. Other statistical approaches based on Dirichlet mixture model are shown to be well suited for single cell clustering, especially for data as unique molecular identifiers (UMI) matrix. For example, DIMM-SC models UMI count data and characterizes variations across different cell clusters via a Dirichlet mixture prior [18]. Para-DPMM further improves the clustering quality by introducing a Dirichlet process prior to automatically infers the number of clusters from the dataset, and a split-merge mechanism is adopted to improve convergence and optimality of the result [19]. Given the high dimensionality of single-cell data, clustering directly on the original dimension may affect the performance due to the intrinsic noise of single-cell data, and usually demands tedious preprocessing such feature selection, which may restrict the robustness of clustering. A simple idea is to use traditional methods to reduce dimensions and then cluster. However, there are too many alternative combinations that make it difficult for us to make a choice. Methods combining dimension reduction with classic clustering such as t-Distributed Stochastic Neighbor Embedding (t-SNE) with K-means [8], and principal component analysis (PCA) with hierarchical clustering [20] for single-cell clustering are proposed. Combining PCA with K-means, a consensus clustering approach is proposed to achieve high accuracy and robustness [21]. CIDR further uses an implicit imputation approach to alleviate the impact of dropouts in scRNA-seq data in a principled manner and identifies putative cell types using hierarchical clustering [22]. Other recent works are also proposed to cluster high dimensional single-cell data with gene regulatory networks [23], or ranking on shared nearest neighbors (SNN) [13].

However, there are still limitations in single-cell clustering to be addressed. First, even the state-of-the-art methods have achieved encouraging performance, the clustering quality can still be significantly improved for challenging tasks as shown in the Results Section. Second, most methods are designed for one of the continuous data and discrete UMI counts. There is demand for methods that can be applied to single-cell data created with different scRNA-seq technologies, such as discrete counts created with UMI based techniques and continuous data normalized to transcripts per million mapped reads (TPM)—if reads are only generated from one end of the transcript, or fragments per kilobase per million mapped reads (FPKM)—if reads cover the entire transcript [24]. Third, a method that can be generally applied to data of various sizes or dimensions is desirable. Nevertheless, most proposed methods cannot scale up well with various data dimensionality and with different dataset size. Last but equally important, most public packages cannot be easily installed due to system environment or software version problems. In addition, some of them require other dependency packages, and thus further inconveniences the use of these packages. Therefore, there is a demand for an easy-to-install and easy-to-use package.

Motivated by the above understanding, we propose in this paper a model-based algorithm named VPAC (Variational Projection for Accurate Clustering) of single-cell transcriptomic data. VPAC is a novel extension of the framework of probabilistic principal components analysis (PPCA) [25], which has been shown to be effective in dimensionality reduction for scRNA-seq data [26]. VPAC projects single-cell samples to a latent space, where the samples are constrained to follow a Gaussian mixture distribution [27]. With a coordinate ascent variational inference (CAVI) algorithm, VPAC can implement parameter estimation efficiently and steadily. Using five scRNA-seq datasets, we show that our model is not only superior to existing methods in the clustering of single-cell transcriptomic data, but also able to be applied to single-cell data of both discrete counts and normalized continuous data. Through comprehensive experiments, we further show the robustness of our model for various data dimensionality, different dataset size and different data sparsity, and the ability of our model to detect genes with strong unique signatures of a specific cell type.

## Methods

### The statistical model of VPAC

Let *N* be the number of single-cell samples, *D* the number of genes, and *M* the desired number of clusters. We assume that the gene expression vector **x**_**n**_ of cell *n* is generated from a projection of a latent *L*-dimensional vector **z**_**n**_ (*L* ≪ *D*). We use *n* = 1, ..., *N* to index over samples, *i* = 1, ..., *L* to index over latent dimensions, and *j* = 1, …, *M* to index over clusters in all derivations below. The distribution of **x**_**n**_ could be a complex high-dimensional distribution. We assume **x**_**n**_ follows a multivariate Gaussian distribution given **z**_**n**_, while the latent vector **z**_**n**_ follows a Gaussian mixture distribution. An intuitive understanding of this assumption is that, when projecting, constraining samples to follow a Gaussian mixture distribution in the latent space contributes to the accurate clustering. As a generative model, VPAC independently draw each sample **x**_**i**_ through the following process.

1 $$ P\left({\mathbf{z}}_n|\mathbf{m},\mathbf{T},\mathbf{s}\right)=\prod \limits_{j=1}^M\mathcal{N}{\left({\mathbf{z}}_n|{\mathbf{m}}_j,{\mathbf{T}}_j^{-1}\right)}^{s_{jn}} $$

2 $$ P\left({\mathbf{x}}_{\mathbf{n}}|{\mathbf{z}}_{\mathbf{n}},\mathbf{W},\boldsymbol{\mu}, \tau \right)=\mathcal{N}\left({\mathbf{x}}_{\mathbf{n}}|{\mathbf{Wz}}_{\mathbf{n}}+\boldsymbol{\mu}, {\tau}^{-1}{\mathbf{I}}_{\mathrm{D}}\right) $$

In the Gauss mixture distribution (1), the binary latent variable s_*jn*_, which gives discrete distributions governed by ***ρ*,** describes which component in the mixture gives rise to the latent vector **z**_**n**_, i.e., if **z**_**n**_ is generated from component *j* then s_*jn*_ = 1, and s_*jn*_ = 0 otherwise. We choose a Dirichlet distribution for the prior of ***ρ***

$$ P\left(s\left|\rho \right.\right)=\prod \limits_{i=j}^M\prod \limits_{n=1}^N{\rho_j}^{s_{jn}}P\left(\rho \right)=\kern0.5em \mathrm{Dir}\left({\rho}_1\cdots {\rho}_M|{\delta}_1\cdots {\delta}_M\right) $$

We complete the specification of Gaussian mixture distribution in Eq. (1) by introducing Gaussian and Wishart conjugate priors over the means **m** and precisions **T**, as

$$ P\left(\boldsymbol{m}\right)=\prod \limits_{j=1}^M\mathcal{N}\left({\mathbf{m}}_j|0,{\gamma}^{-1}\mathbf{I}\right)P\left(\mathbf{T}\right)=\kern0.5em \prod \limits_{j=1}^M\mathcal{W}\left({\mathbf{T}}_j|\upsilon, \mathrm{V}\right) $$

where *γ* is a small and fixed parameter chosen to give a broad prior to **m**, while the degrees of freedom *υ* and scale matrix V give a broad prior to **T**.

In order to endow VPAC the ability to automatically determine the appropriate dimensionality for the latent space to avoid discrete model selection, we introduce a hierarchical prior *P*(**W**| ***α***) over the projection matrix **W** in Eq. (2), as

$$ P\left(\mathbf{W}|\boldsymbol{\alpha} \right)=\prod \limits_{i=1}^L\mathcal{N}\left({\mathbf{W}}_i|\mathbf{0},{\alpha}_i^{-1}{\mathbf{I}}_D\right) $$

where each item of the *L*-dimensional vector ***α*** controls the corresponding column of the matrix **W** by playing a role as the precision of a Gaussian distribution. We again introduce broad priors over the parameters ***α,μ*** and *τ* to complete the specification of VPAC

$$ P\left(\boldsymbol{a}\right)=\prod \limits_{i=1}^L\Gamma \left({\alpha}_i|{a}_{\alpha },{b}_{\alpha}\right)P\left(\boldsymbol{\mu} \right)=\mathcal{N}\left(\boldsymbol{\mu} |0,{\beta}^{-1}\mathbf{I}\right)P\left(\tau \right)=\Gamma \left(\tau |{a}_{\tau },{b}_{\tau}\right) $$

**Fig. 1:**
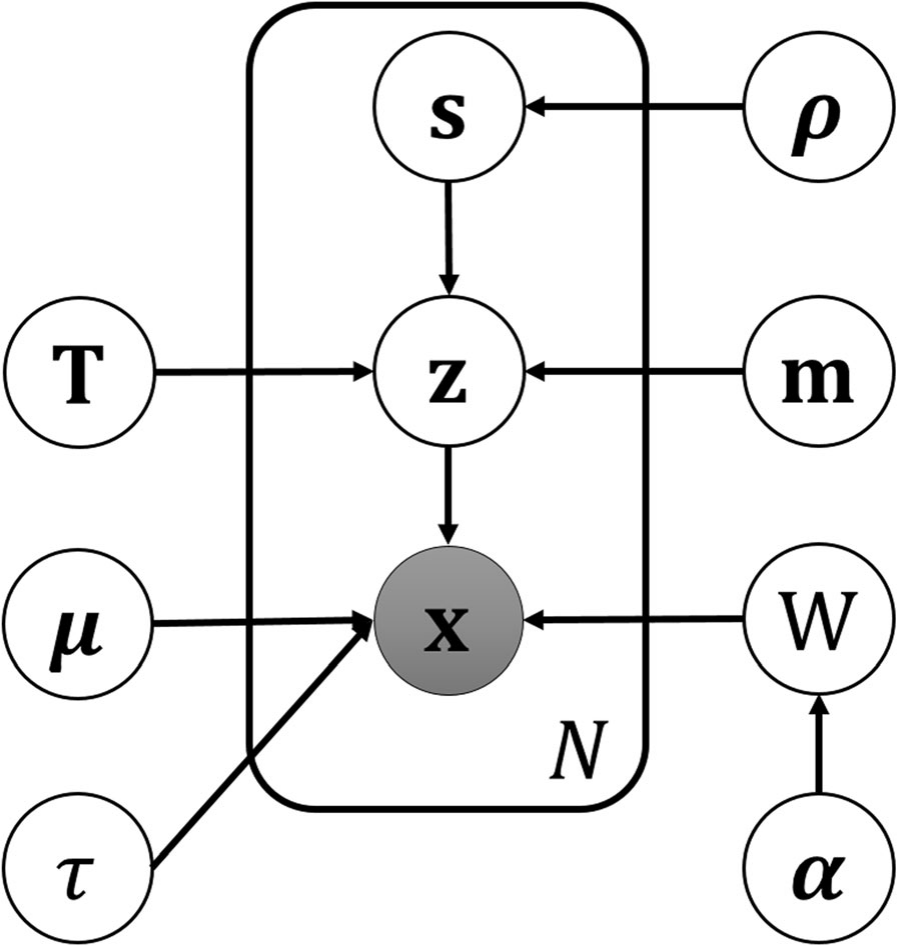
Representation of VPAC as a probabilistic graphical model. The observed variable x is shown by the shaded node, while the plate notation comprises a dataset of *N* independent observations together with the corresponding latent variables

The graphical model representation of VPAC is shown in Fig. 1. The broad priors introduced above are obtained by setting *a*_*α*_ = *b*_*α*_ = *a*_*τ*_ = *b*_*τ*_ = *β* = *γ* = 10^−3^. The initial parameters ***δ*** of the Dirichlet distribution are set as $\frac{1}{M}$. The joint distribution of all of the variables is given by

$$ P\left(\boldsymbol{\rho}, \mathbf{s},\mathbf{T},\mathbf{m},\mathbf{Z},\tau, \boldsymbol{\mu}, \boldsymbol{\alpha}, \mathbf{W},\mathbf{X}\right)=\prod \limits_{n=1}^N\left[P\left(\boldsymbol{\rho} \right)P\left(\mathbf{s}|\boldsymbol{\rho} \right)P\left(\mathbf{T}\right)P\left(\mathbf{m}\right)P\left({\mathbf{z}}_n|\mathbf{m},\mathbf{T},\mathbf{s}\right)\right]\left[P\left(\tau \right)P\left(\boldsymbol{\mu} \right)P\left(\boldsymbol{\alpha} \right)P\left(\mathbf{W}|\boldsymbol{\alpha} \right)P\left({\mathbf{x}}_{\mathrm{n}}|{\mathbf{z}}_{\mathrm{n}},\mathbf{W},\boldsymbol{\mu}, \tau \right)\right] $$

### Variational inference of parameters

We use variational methods to find a lower bound on *P*(**X**) because it is analytically intractable to directly evaluate *P*(**X**). With ***θ*** denoting the set of all parameters and latent variables in VPAC, we introduce an approximating distribution *Q*(***θ***) of the true posterior distribution. The log marginal likelihood is then given by

$$ \ln P\left(\mathbf{X}\right)=\ln \int P\left(\mathbf{X},\boldsymbol{\theta} \right)d\boldsymbol{\theta} =\ln \int Q\left(\boldsymbol{\theta} \right)\frac{P\left(\mathbf{X},\boldsymbol{\theta} \right)}{Q\left(\boldsymbol{\theta} \right)}d\boldsymbol{\theta} \ge \int Q\left(\boldsymbol{\theta} \right)\ln \frac{P\left(\mathbf{X},\boldsymbol{\theta} \right)}{Q\left(\boldsymbol{\theta} \right)}d\boldsymbol{\theta} =\mathrm{\mathcal{L}}(Q) $$

The function $\mathcal{L}(Q)$ is the evidence lower bound (ELBO) on the true log marginal likelihood. The goal is to find a suitable *Q*(***θ***) to maximize the ELBO or minimize the Kullback-Leibler divergence between $\mathcal{L}(Q)$ and the true log marginal likelihood

$$ KL\left(Q\parallel P\right)=-\int Q\left(\boldsymbol{\theta} \right)\ln \frac{P\left(\boldsymbol{\theta} |\mathbf{X}\right)}{Q\left(\boldsymbol{\theta} \right)}d\boldsymbol{\theta} $$

We assume the variational approximation is mean-field, i.e., $Q\left(\boldsymbol{\theta} \right)=\prod \limits_t{Q}_t\left({\theta}_t\right)$. The corresponding factors are

$$ Q\left(\mathbf{z}\right)=\prod \limits_{n=1}^N\mathcal{N}\left(\tilde{{\mathbf{z}}_{\boldsymbol{n}}}|{\mathbf{m}}_{\boldsymbol{z}}^{\left(\boldsymbol{n}\right)},{\boldsymbol{\Sigma}}_{\boldsymbol{z}}^{\left(\boldsymbol{n}\right)}\right)Q\left(\mathbf{W}\right)=\prod \limits_{i=1}^L\mathcal{N}\left(\tilde{{\mathbf{w}}_{\boldsymbol{i}}}|{\mathbf{m}}_{\boldsymbol{W}}^{\left(\boldsymbol{i}\right)},{\boldsymbol{\Sigma}}_{\boldsymbol{W}}\right)Q\left(\boldsymbol{\mu} \right)=\mathcal{N}\left(\tilde{\boldsymbol{\mu}}|{\mathbf{m}}_{\boldsymbol{\mu}},{\boldsymbol{\Sigma}}_{\boldsymbol{\mu}}\right)Q\left(\boldsymbol{\alpha} \right)=\prod \limits_{i=1}^L\Gamma \left(\tilde{{\boldsymbol{\alpha}}_i}|\tilde{a_{\alpha }},\tilde{b_{\alpha i}}\right)\ Q\left(\tau \right)=\Gamma \left(\tilde{\tau}|\tilde{a_{\tau }},\tilde{b_{\tau }}\right)Q\left(\boldsymbol{\rho} \right)=\mathrm{Dir}\left(\tilde{\rho_1}\cdots \tilde{\rho_M}\right)Q\left(\boldsymbol{s}\right)=\prod \limits_{n=1}^N\prod \limits_{j=1}^M{\mathbf{P}}_{jn}^{s_{jn}}Q\left(\mathbf{m}\right)=\prod \limits_{j=1}^M\mathcal{N}\left(\tilde{{\mathbf{m}}_{\boldsymbol{j}}}|{\mathbf{m}}_{\mathbf{m}}^{\left(\boldsymbol{j}\right)},{\boldsymbol{\Sigma}}_{\mathbf{m}}^{\left(\boldsymbol{j}\right)}\right)Q\left(\mathbf{T}\right)=\prod \limits_{j=1}^M\mathcal{W}\left(\tilde{{\mathbf{T}}_j}|{\upsilon}_T^{(j)},{\mathbf{V}}_{\mathrm{T}}^{(j)}\right) $$

Because the proposed model is conjugate, we can derive a coordinate ascent variational inference (CAVI) algorithm to update the variational parameters

$$ {\boldsymbol{\Sigma}}_{\mathbf{z}}^{(n)}={\left(\left\langle \tau \right\rangle \left\langle {\mathbf{W}}^{\mathrm{T}}\mathbf{W}\right\rangle +\sum \limits_{j=1}^M\left\langle {\mathrm{s}}_{jn}\right\rangle \left\langle {\mathbf{T}}_j\right\rangle \right)}^{-1}{\mathbf{m}}_{\mathbf{z}}^{(n)}=\left({\left({\mathbf{x}}_n-\left\langle \boldsymbol{\mu} \right\rangle \right)}^{\mathrm{T}}\left\langle \tau \right\rangle \left\langle \mathbf{W}\right\rangle +\sum \limits_{j=1}^M\left\langle {\mathrm{s}}_{jn}\right\rangle \left\langle {\mathbf{m}}_j^{\mathrm{T}}\right\rangle \left\langle {\mathbf{T}}_j\right\rangle \right){\boldsymbol{\Sigma}}_{\mathbf{z}}^{(n)}{\boldsymbol{\Sigma}}_{\mathbf{W}}={\left(\operatorname{diag}\left(\left\langle \boldsymbol{\alpha} \right\rangle \right)+\left\langle \tau \right\rangle \sum \limits_{n=1}^N\left\langle {\mathbf{z}}_n^{\mathrm{T}}{\mathbf{z}}_n\right\rangle \right)}^{-1}{\mathbf{m}}_{\mathbf{W}}^{(i)}=\left\langle \tau \right\rangle {\boldsymbol{\Sigma}}_{\mathbf{W}}\sum \limits_{n=1}^N\left\langle {\mathbf{z}}_n\right\rangle \left({\mathrm{x}}_{ni}-\left\langle {\mu}_i\right\rangle \right){\boldsymbol{\Sigma}}_{\boldsymbol{\mu}}={\left(\beta +N\left\langle \tau \right\rangle \right)}^{-1}\mathbf{I}{\mathbf{m}}_{\boldsymbol{\mu}}={\boldsymbol{\Sigma}}_{\mu}\sum \limits_{n=1}^N{\left({\mathbf{x}}_n-\left\langle \mathbf{W}\right\rangle \left\langle {\mathbf{z}}_n\right\rangle \right)}^{\mathrm{T}}\left\langle \tau \right\rangle\ \tilde{a_{\alpha }}={a}_{\alpha }+\frac{D}{2}\tilde{b_{\alpha i}}={b}_{\alpha }+\frac{1}{2}\left\langle {\left\Vert {\mathbf{W}}_i\right\Vert}^2\right\rangle\ \tilde{a_{\tau }}={a}_{\tau }+\frac{ND}{2}\tilde{b_{\tau }}={b}_{\tau }+\frac{1}{2}\sum \limits_{n=1}^N\left\{{\left\Vert {\mathbf{x}}_n\right\Vert}^2+{\left\Vert \boldsymbol{\mu} \right\Vert}^2+\mathrm{Tr}\left(\left\langle {\mathbf{W}}^{\mathrm{T}}\mathbf{W}\right\rangle \left\langle {\mathbf{z}}_n{\mathbf{z}}_n^{\mathrm{T}}\right\rangle \right)+2\left\langle {\boldsymbol{\mu}}^{\mathrm{T}}\right\rangle \left\langle \mathbf{W}\right\rangle \left\langle {\mathbf{z}}_n\right\rangle -2{\mathbf{x}}_n^{\mathrm{T}}\left\langle \mathbf{W}\right\rangle \left\langle {\mathbf{z}}_n\right\rangle -2{\mathbf{x}}_n^{\mathrm{T}}\left\langle \boldsymbol{\mu} \right\rangle \right\}\ \tilde{\rho_j}={\rho}_j+\sum \limits_{n=1}^N{s}_{jn}\ {\mathbf{P}}_{jn}=\frac{\tilde{{\mathrm{P}}_{jn}}}{\sum_{l=1}^M\tilde{P_{ln}}}\tilde{{\mathbf{P}}_{jn}}=\exp \left\{\frac{\ln \left|{\mathbf{T}}_j\right|}{2}+\ln \left\langle {\rho}_j\right\rangle -\frac{1}{2}\mathrm{Tr}\left[\left\langle {\mathbf{T}}_j\right\rangle \left(\left\langle {\mathbf{z}}_n{\mathbf{z}}_n^T\right\rangle -\left\langle {\mathbf{m}}_j\right\rangle {\left\langle {\mathbf{z}}_n\right\rangle}^T-\left\langle {\mathbf{z}}_n\right\rangle {\left\langle {\mathbf{m}}_j\right\rangle}^T+\left\langle {\mathbf{m}}_j{\mathbf{m}}_j^T\right\rangle \right)\right]\right\}\ {\boldsymbol{\Sigma}}_{\mathbf{m}}^{(j)}={\left(\gamma \mathbf{I}+\left\langle {\mathbf{T}}_j\right\rangle \sum \limits_{n=1}^N\left\langle {\mathrm{s}}_{jn}\right\rangle \right)}^{-1}{\mathbf{m}}_{\mathbf{m}}^{(j)}={\boldsymbol{\Sigma}}_m^{(j)}\left\langle {\mathbf{T}}_j\right\rangle \sum \limits_{n=1}^N\left\langle {\mathrm{s}}_{jn}\right\rangle {\mathrm{z}}_{jn}\ {\upsilon}_{\boldsymbol{T}}^{(j)}=\upsilon +\sum \limits_{n=1}^N\left\langle {\mathrm{s}}_{jn}\right\rangle {\mathbf{V}}_{\boldsymbol{T}}^{(j)}=\mathbf{V}+\sum \limits_{n=1}^N\left[\left\langle {\mathbf{z}}_n{\mathbf{z}}_n^{\mathrm{T}}\right\rangle \left\langle {\mathrm{s}}_{jn}\right\rangle -\left\langle {\mathbf{z}}_n\right\rangle \left\langle {\mathrm{s}}_{jn}\right\rangle \left\langle {\mathbf{m}}_j^{\mathrm{T}}\right\rangle -\left\langle {\mathbf{m}}_j\right\rangle \left\langle {\mathbf{z}}_n^{\mathrm{T}}\right\rangle \left\langle {\mathrm{s}}_{jn}\right\rangle +\left\langle {\mathbf{m}}_j{\mathbf{m}}_j^{\mathrm{T}}\right\rangle \left\langle {\mathrm{s}}_{jn}\right\rangle \right] $$

We implement VPAC in Python using common packages for data analysis (including Numpy, Scikit-learn and Scipy), without other dependency packages. Users can directly import our VPAC class and conduct clustering. Algorithm 1 gives a pseudo-code description of VPAC.

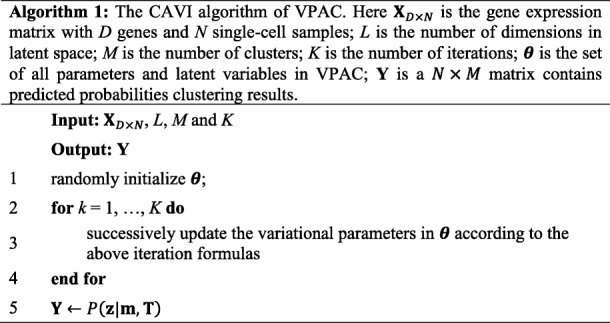



### Data collection

We collected a dataset of three T cell types (CD4+/CD25+ regulatory T cells, CD4+/CD45RA+/CD25- naive T cells and CD8+/CD45RA+ naïve cytotoxic T cells) from 10X Genomics [28]. The dataset measures the expression of 32,738 genes in 32,695 cells, which were enriched from fresh peripheral blood mononuclear cells (PBMCs) and sequenced by Illumina NextSeq 500 instrument. Clustering on this dataset was claimed as a challenging task by Z.Sun et al. [18] and T.Duan et al. [19]. We used this dataset to demonstrate the ability of our method to scale up well with various data dimensionality and with different dataset size on datasets from UMI based techniques. We also downloaded three preprocessed datasets of different scales used by Para-DPMM [19], which is the state-of-the-art method for clustering discrete UMI counts, to fairly evaluate the performance of our model.

A dataset of 561 cells derived from seven cell lines (A549, H1437, HCT116, IMR90, K562, GM12878, and H1) was downloaded from the NCBI Gene Expression Omnibus via accession GSE81861 [29]. Sequenced using the 101-bp paired-end protocol on the Illumina HiSeq 2000 platform, the dataset measures the expression of 55,186 DNA regions, and the expression was quantified as FPKM values. We used this dataset to demonstrate the robustness of our model for FPKM-normalized data of various dimensions. From the Single Cell Portal, we downloaded a plate-based scRNA-seq dataset of 24,649 genes in 27,998 cells from 12 clusters. The dataset was sequenced using a modified Smart-seq2 protocol, and the expression levels of genes were quantified as TPM values [30]. With this dataset, we further demonstrated the robustness of our model for TPM-normalized datasets of different size.

In order to illustrate the superior ability of our model to account for the sparsity of scRNA-seq data, we collected a dataset of 301 cells captured from 11 cell types using microfluidics. This dataset is publicly available from the NCBI Sequence Read Archive under accession SRP041736, and the quantification of gene expression levels in TPM for all 23,730 genes in all samples was performed [31]. We also collected a Smart-seq2 sequenced dataset of 742 dendritic cells from six clusters with two batches from the Single Cell Portal to illustrate the application of our model [32, 33].

### Assessment of performance

We used adjusted rand index (ARI) [34] and normalized mutual information (NMI) [35] to assess the clustering performance, namely, the similarity between the clustering results and known cell types provided by their original references. Suppose T is the known cell types, C the predicted clustering results, *N* the total number of single-cell samples, *x*_*i*_ the number of samples clustered to the *i*-th cluster of C, *y*_*j*_ the number of samples belong to the *j*-th cell type of T, and *n*_*ij*_ the number of overlapping samples between the *i*-th cluster and the *j*-th cell type. ARI is computed as

$$ \mathrm{ARI}=\frac{\sum \limits_{ij}\left(\genfrac{}{}{0pt}{}{n_{ij}}{2}\right)-\left[\sum \limits_i\left(\genfrac{}{}{0pt}{}{x_i}{2}\right)\sum \limits_j\left(\genfrac{}{}{0pt}{}{y_j}{2}\right)\right]/\left(\genfrac{}{}{0pt}{}{N}{2}\right)\ }{\frac{1}{2}\left[\sum \limits_i\left(\genfrac{}{}{0pt}{}{x_i}{2}\right)+\sum \limits_j\left(\genfrac{}{}{0pt}{}{y_j}{2}\right)\right]-\left[\sum \limits_i\left(\genfrac{}{}{0pt}{}{x_i}{2}\right)\sum \limits_j\left(\genfrac{}{}{0pt}{}{y_j}{2}\right)\right]/\left(\genfrac{}{}{0pt}{}{N}{2}\right)} $$

where ()denotes a binomial coefficient. By denoting the entropy of C and T as H(C) and H(T), respectively, and the mutual information between them as MI(C,T), NMI can be computed as

$$ \mathrm{NMI}\left(\mathrm{C},\mathrm{T}\right)=\frac{\mathrm{MI}\left(\mathrm{C},\mathrm{T}\right)}{\sqrt{\mathrm{H}\left(\mathrm{C}\right)\mathrm{H}\left(\mathrm{T}\right)}} $$

## Results

### VPAC accurately clusters scRNA-seq data

To verify the clustering performance of VPAC, we conducted each experiment five times and computed two widely used metrics, ARI and NMI. We compared the performance of our method with four baseline methods (using their default parameters), including Para_DPMM, the state-of-the-art method for clustering discrete UMI counts [19], pcaReduce, another method combining dimension reduction with clustering [20], and other two widely used methods (Seurat [13] and SC3 [21]).

In order to fairly evaluate the performance of our model, we compared the performance of VPAC with other four baseline methods on the three T cell datasets of different scales provided by T.Duan et al. [19]. The small scale dataset (S-Set) consists of 1200 cells with the 1000 top variable genes, the medium scale dataset (M-Set) consists of 3000 cells with the 3000 top variable genes, and the large scale dataset (L-Set) consists of 6000 cells with the 5000 top variable genes. As shown in Table 1, our method consistently outperforms the four baseline methods for a large margin on all the three datasets. In more detail, pcaReduce and Seurat fail in the clustering task, while SC3 achieves satisfactory performance in the first two datasets. However, the performance of SC3 decreases dramatically when the number of cells exceeds about 3 thousand, which will be further demonstrated in the following sections. Compared to Para_DPMM, our method achieves average 11.3% improvement on ARI and 8.1% improvement on NMI even the clustering on these datasets was claimed as a challenging task [18, 19]. In addition, our method consistently achieves stable performance, which demonstrates its higher robustness than the baseline methods. In terms of efficiency, the performance of VPAC is moderate compared with discriminant methods, which have an inherent advantage of high speed. Taking the L-Set as an example, VPAC cost 241 s, Para_DPMM cost 1000 s, pcaReduce cost 37 s, Seurat cost 66 s and SC3 cost 653 s on average. It is worth noting that the default minimal training time of Para_DPMM is 1000 s, which slightly improves the performance of Para_DPMM shown in the original research.

**Table 1:** Performance comparison on three datasets of different scales

Dataset	S-Set		M-Set		L-Set	
Model	ARI	NMI	ARI	NMI	ARI	NMI
VPAC	0.769 ± 0.000	0.759 ± 0.000	0.765 ± 0.000	0.765 ± 0.000	0.779 ± 0.000	0.769 ± 0.000
Para_DPMM	0.675 ± 0.008	0.696 ± 0.008	0.704 ± 0.000	0.713 ± 0.002	0.700 ± 0.002	0.711 ± 0.002
pcaReduce	0.290 ± 0.019	0.289 ± 0.010	0.279 ± 0.007	0.252 ± 0.007	0.311 ± 0.026	0.309 ± 0.044
Seurat	0.378 ± 0.000	0.379 ± 0.000	0.379 ± 0.000	0.383 ± 0.000	0.383 ± 0.000	0.383 ± 0.000
SC3	0.724 ± 0.005	0.722 ± 0.010	0.724 ± 0.009	0.717 ± 0.007	0.523 ± 0.053	0.501 ± 0.030

### VPAC scales up well with various data dimensionality

**Fig. 2:**
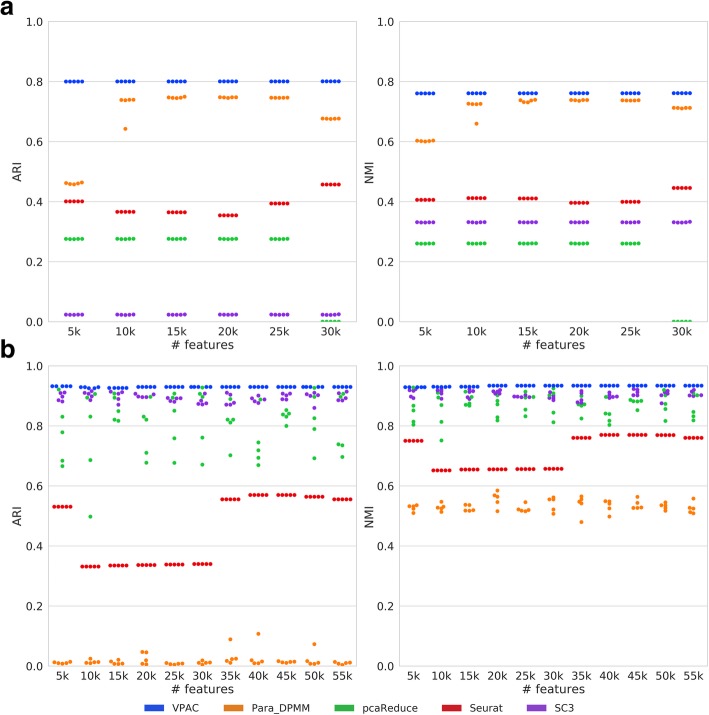
Performance comparison on datasets of various dimensionality. **a** The performance on discrete UMI counts. **b** The performance on continuous FPKM-normalized data

To illustrate the robustness of our model for various data dimensionality, we selected different numbers of top variable genes (features) based on their standard deviations across the cell transcriptome profiles. We first used the dataset of 32,695 cells enriched from PBMCs, which is the full version of the three preprocessed datasets, to demonstrate the superior performance of VPAC for discrete UMI counts of various dimensionality. As illustrated in Fig. 2a, our method significantly outperforms the four baseline methods and achieves consistent performance across datasets of different numbers of features. Note that pcaReduce raised an error when training the dataset with 30 k features even the memory is sufficient. SC3 cannot be competent for clustering the dataset of 32,695 cells.

We further evaluated the robustness of our model for FPKM-normalized data using a dataset of 561 cells derived from seven cell lines. As shown in Fig. 2b, VPAC consistently outperforms the four baseline methods again. Different from the performance for discrete UMI counts, all the performance of pcaReduce, Seurat and SC3 are superior to that of Para_DPMM. SC3 achieves the second best performance, but, as other three baseline methods, the performance is obviously unstable, while VPAC achieves consistent performance across datasets of different numbers of features. The results demonstrate that VPAC can scale up well with various data dimensionality whether the scRNA-seq data is discrete or continuous.

### VPAC scales up well with different dataset size

**Fig. 3:**
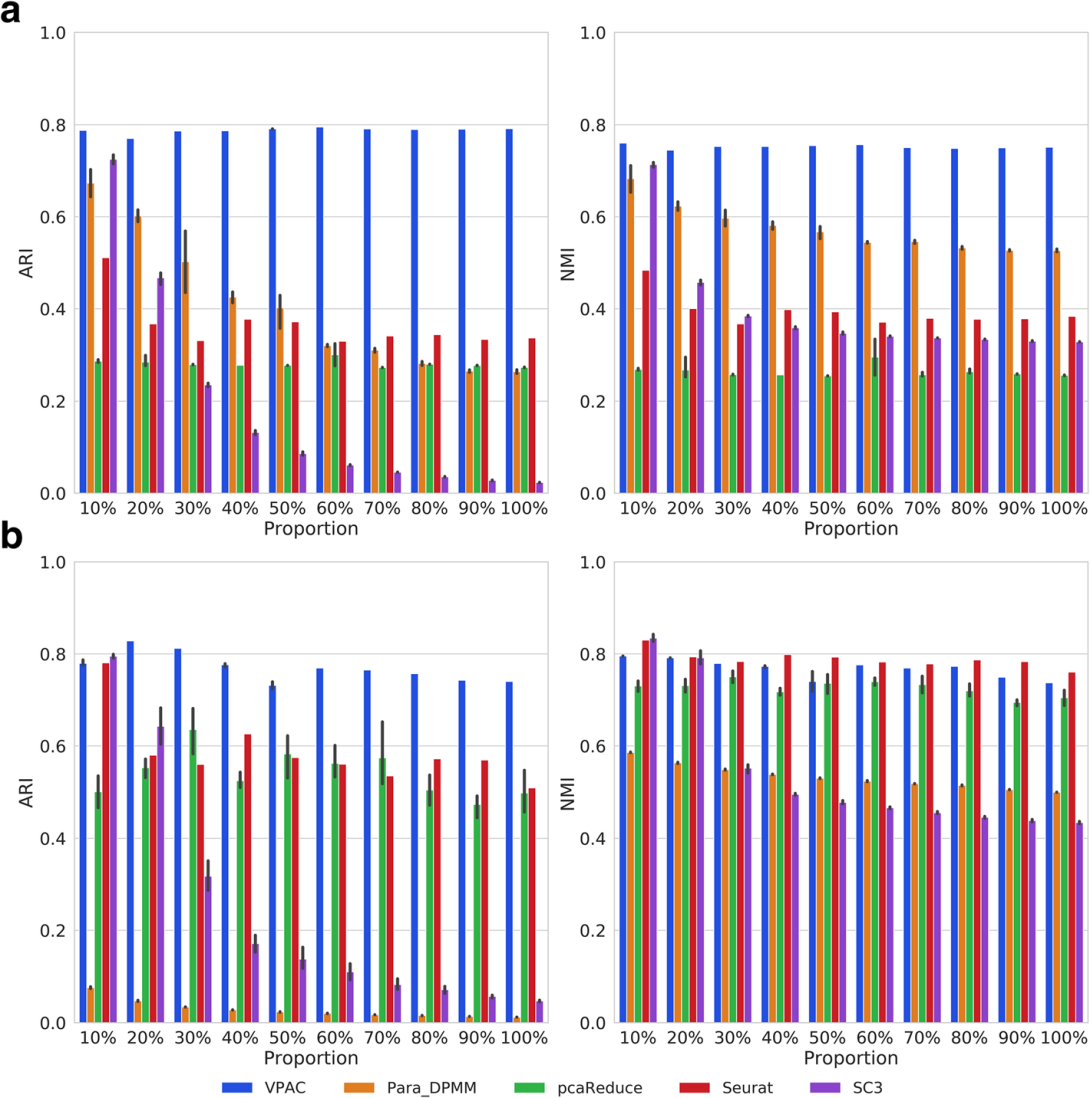
Performance comparison on datasets of different size. **a** The performance on discrete UMI counts. **b** The performance on continuous TPM-normalized data

Different scRNA-seq technologies can be used to quantify transcriptomes for different numbers of cells. In order to demonstrate the robustness of our model for different dataset size, we randomly selected different proportions of single-cell samples from the whole cell population. Besides, we selected the top 90% variable genes (features) to avoid errors raised by pcaReduce. We again used the cells enriched from PBMCs to illustrate the superior performance of VPAC for discrete UMI counts of different dataset size. As illustrated in Fig. 3a, our method significantly outperforms the four baseline methods and achieves superior performance across datasets of different numbers of samples. As the number of samples increased, the performance of Para_DPMM deteriorates, while that of SC3 decreases dramatically since the number of cells exceeds about 3 thousand, which confirms the previous results.

We further illustrated the robustness of our model for TPM-normalized data using a dataset of 24,649 cells from 12 clusters. As shown in Fig. 3b, VPAC is still superior to the baseline methods and achieves more stable performance across datasets of different numbers of samples. The ARI scores of VPAC are higher than that of Seurat for a large margin, even that Seurat achieves comparable NMI scores. The performance of SC3 again deteriorates as the number of samples increased. The performance of pcaReduce is still very unstable even outperforms Para_DPMM. The above results demonstrate that VPAC can also scale up well with different dataset size whether the scRNA-seq data is discrete or continuous.

### VPAC scales up well with different data sparsity

**Fig. 4:**
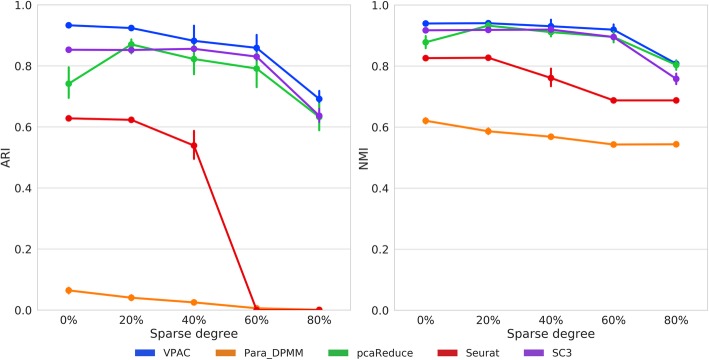
Performance comparison on datasets of different sparsity

Single-cell gene expression data suffer from the high sparsity problem because they contain an abundance of dropout events that lead to zero expression measurements. In order to illustrate the superior ability of our model to account for the sparsity of scRNA-seq data, we randomly zeroed different proportions of non-zero items five times on a TPM-normalized dataset of 301 cells with about 2.7 × 10^5^ reads per cell [31]. As illustrated in Fig. 4, VPAC is superior to the baseline methods and achieves stable performance. One-sided paired-sample Wilcoxon signed rank test consistently suggest that our metty hod achieves higher ARI scores (*p*-values = 5.05e-5 for pcaReduce and 4.98e-5 for SC3) and NMI scores than a baseline method (*p*-values = 8.60e-4 for pcaReduce and 2.25e-5 for SC3). The performance of VPAC, pcaReduce and SC3 obviously deteriorate when we set 80% of the non-zero expression data to zero. Seurat achieves relatively poor performance, while Para_DPMM again fails on clustering the TPM-normalized dataset. The results demonstrate that our model has the ability to account for the sparsity of scRNA-seq data, and thus benefits the clustering tasks with the exponential growth of the Drop-seq based single-cell transcriptomic data.

### Applications of VPAC

**Fig. 5:**
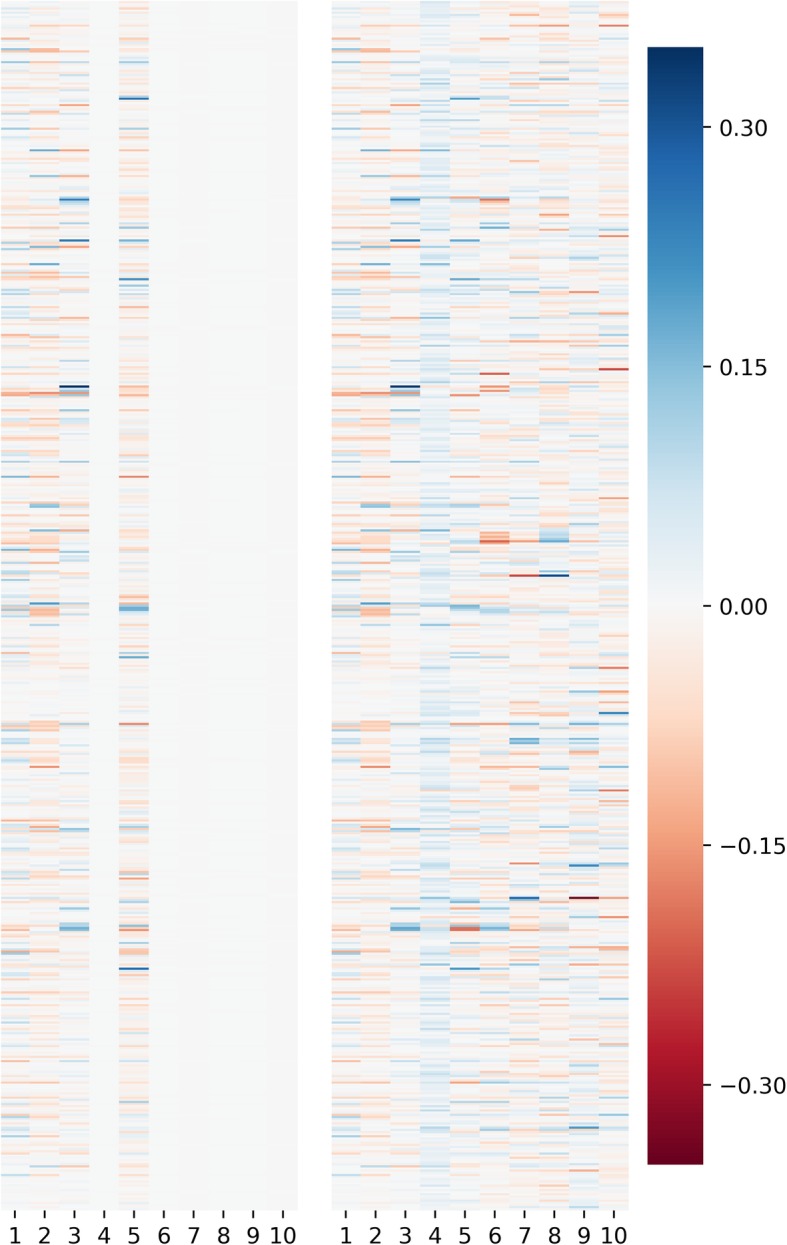
Visualization of the projection matrix W of VPAC (the left one) and that of classical PCA (the right one)

To demonstrate potential applications of VPAC, we collected a dataset of 742 dendritic cells from six clusters [32, 33]. By setting the number of clusters to 6 and the dimensionality of latent space to 10, VPAC achieved an ARI of 0.876 and an NMI of 0.898. Actually, the items of ***α*** with large values in VPAC will result in ‘switching off’ the corresponding column of the matrix **W**, and thus provide guidance for determining the appropriate dimensionality for the latent space. As shown in Fig. 5, the visualization of the projection matrix **W** (the left one) indicates there are four principal directions in the latent space, while the visualization of the projection matrix obtained by classical PCA (the right one) cannot effectively reveal the number of principal directions. Therefore, by setting the dimensionality of latent space to 5 (in practice we have found that one more dimensionality, which may contain some additional information, tends to give better results), VPAC achieved an ARI of 0.875 and an NMI of 0.903, which are approximately equal to the results with the dimensionality of latent space setting to 10, which means the only one parameter we should determine for VPAC is the number of clusters.

**Fig. 6:**
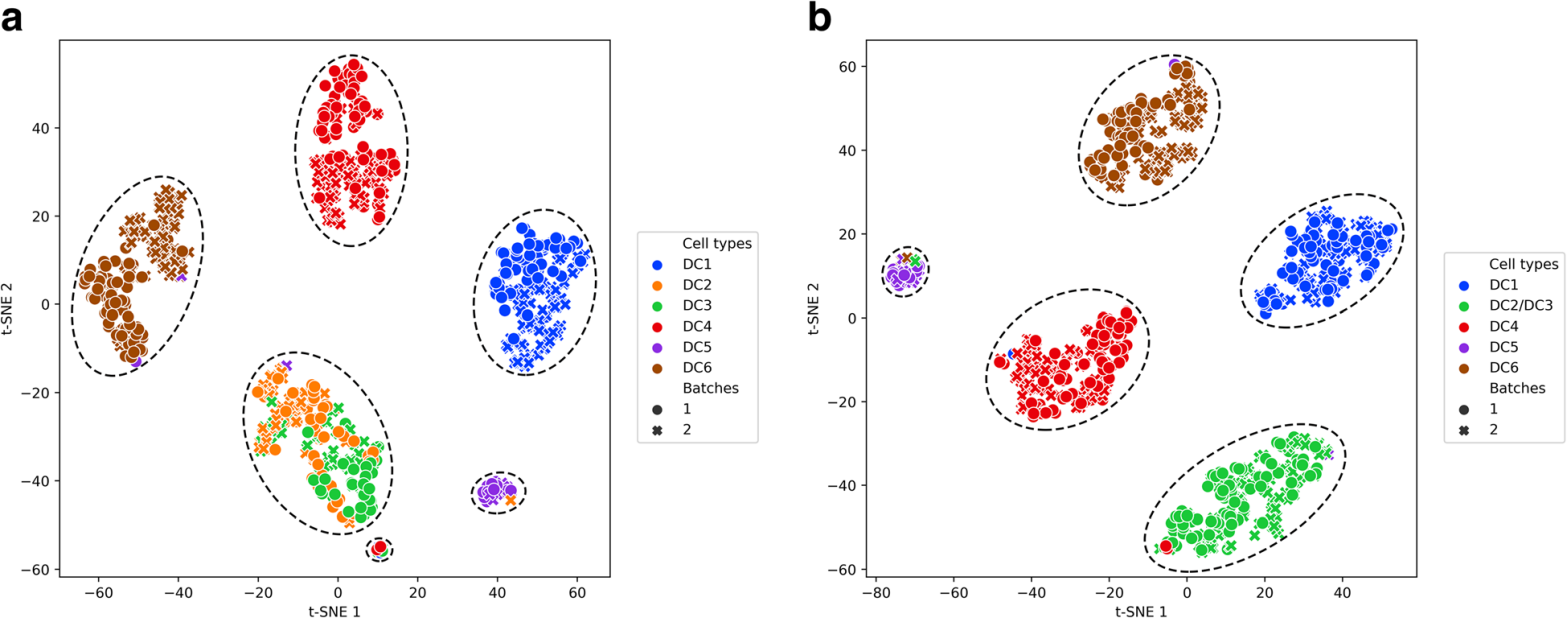
Visualization of the dendritic cells in latent space inferred by VPAC using t-SNE. The dendritic cells are colored by cell-type labels provided by the original study, different shapes of points represent different experimental batches, and the dashed circles represent potential clusters inferred by VPAC with setting the number of clusters to (**a**) 6, and (**b**) 5

We used the t-SNE algorithm to visualize the dimension-reduced data in the latent space of VPAC by projecting the data into a two-dimensional space so that certain hidden structures can be depicted intuitively. Note that t-SNE is a visualization tool, and it is not intended to be used for clustering scRNA-seq data [18]. As shown in Fig. 6a, VPAC accurately clustered different cell types accounting for cells from different batches. It is worth noting that VPAC intended to cluster DC2 and DC3 cell types together while inferring a small cluster (in the bottom) containing some individual cells. According to the original research for this dataset, it is reasonable to cluster DC2 and DC3 together because both of them correspond to new subdivisions of the CD1C/BDCA-1^+^cDC2, while DC1 corresponds to the cross-presenting CD141/BDCA-3^+^ cDC1, DC4 corresponds to CD1C^−^CD141^−^CD11C^+^ DC, DC5 is a unique DC subtype, AS DCs, and DC6 corresponds to the interferon-producing pDC [32]. With this understanding, we set the number of clusters to 5. VPAC successfully clustered the DC2/3 cluster and achieved an ARI of 0.892 and an NMI of 0.943. In addition, as illustrated in Fig. 6b, VPAC accounted for batch effects even there are still very few samples wrongly clustered, which means that our method is robust to scRNA-seq data from different batches.

**Fig. 7:**
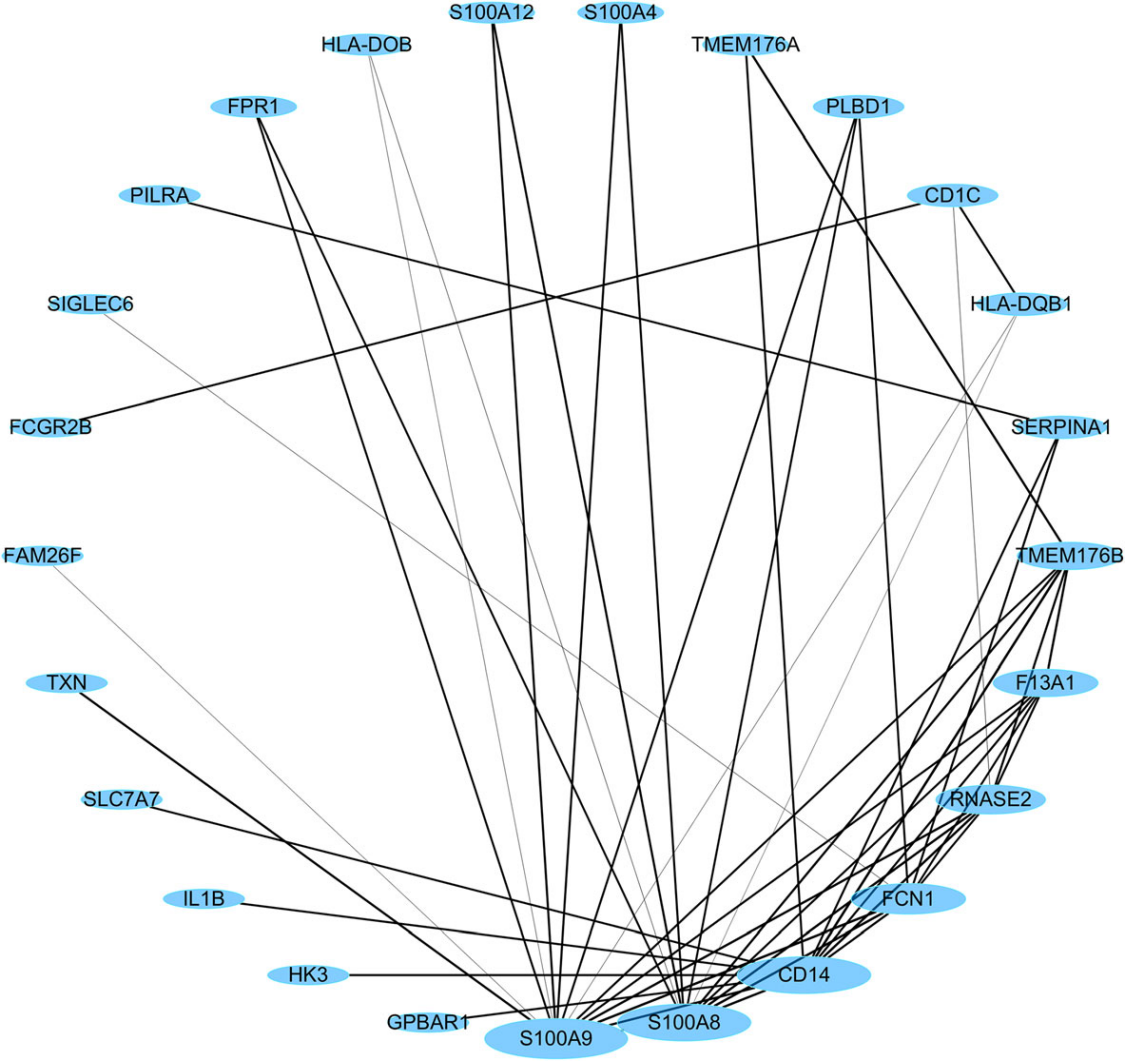
The co-expression network of the DC2/3 cluster inferred by VPAC

We further conducted gene co-expression network analysis to identify which genes have a tendency to show a coordinated expression pattern of the DC2/3 cluster inferred by VPAC. By calculating the Pearson correlation coefficient (PCC) of each gene pairs, we plotted a co-expression network with a threshold 0.4 of PCC [36]. As illustrated in Fig. 7, the three genes, namely S100A9, S100A8 and CD14, with the highest degree of connectivity are expected to be drivers required for signaling pathways of essential functions. It is worth noting that, in the original research, these three genes were claimed as acute and chronic inflammatory genes that play as a strong unique signature to distinguish the DC2/3 cluster, which means our method has the ability to reveal the genes with strong unique signatures of a specific cell type.

**Fig. 8:**
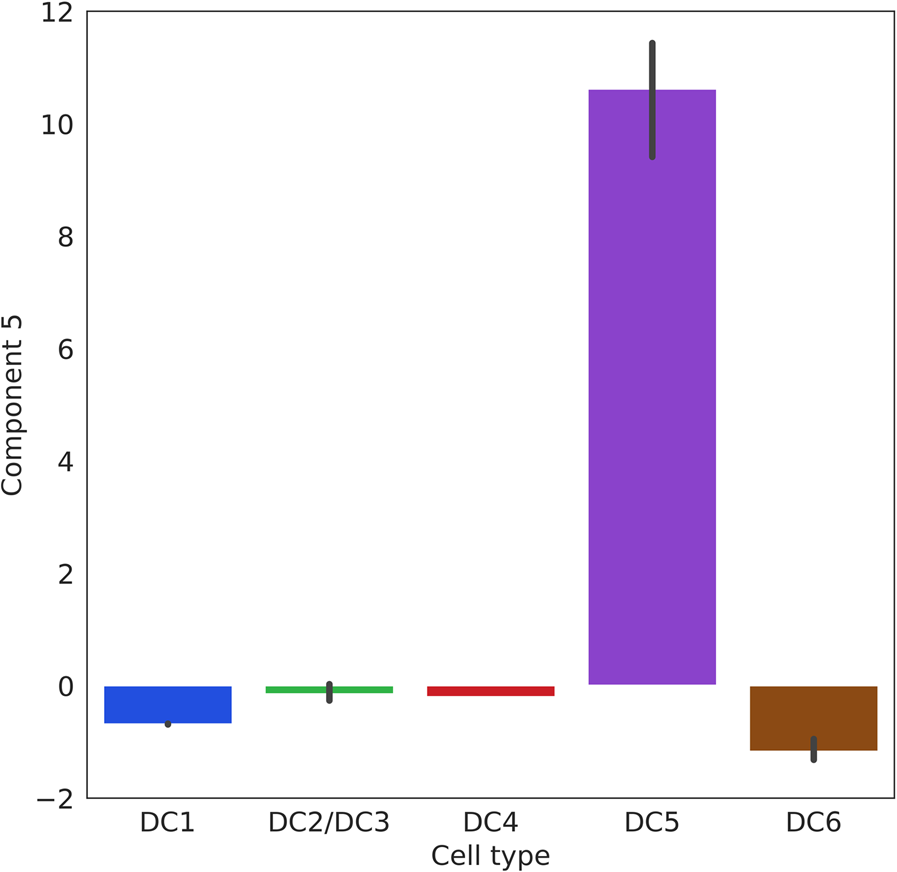
The values of fifth items in the latent vectors inferred by VPAC

It is worth noting that VPAC successfully clustered the novel DC5 samples detected by the original research. In order to study the potential mechanism implied in VPAC, we further analyzed the samples in the latent space inferred by VPAC. As shown in Fig. 8, the fifth items in latent vectors of the DC5 cluster are much larger than that of other clusters, which means that the fifth direction of the latent space can effectively distinguish the DC5 cluster. We sorted the absolute values of the fifth column in projection matrix **W**, which are the absolute weights of linear combinations of genes, and found that the two genes with the highest absolute weights (0.290 and 0.288) are AXL and SIGLEC6, which have much higher absolute weight than the successive gene CX3CR1 with a weight of 0.225. Interestingly, AXL and SIGLEC6 serve as unique markers for the population emerged from the unbiased cluster analysis (cluster DC5) according to the original literature, which further demonstrates that our method has the inherent ability to detect genes with strong unique signatures of a specific cell type while accurately clustering.

## Discussion

scRNA-Seq technologies have advanced rapidly in recent years and enable the quantitative characterization of cell types based on transcriptome profiles. VPAC is proposed to identify putative cell types using unsupervised clustering. The superiority of our method over other baseline methods, such as Para_DPMM and pcaReduce, is mainly attributed to the variational projection while constraining single-cell samples to follow a Gaussian mixture distribution in the latent space. In addition, with the hierarchical prior over the projection matrix, VPAC can automatically determine the appropriate dimensionality for the latent space to avoid discrete model selection, which means the only one parameter we should determine for VPAC is the number of clusters. The comprehensive experiments demonstrate the generality and robustness of our model.

Our model can certainly be improved in some aspects. First, a more complex non-linear projection can be introduced to better model the scRNA-seq data. Second, more constraints or assumptions can be included in our model considering the characteristics of scRNA-seq data, such as the high sparsity. Recent studies have shown the introduction of zero-inflated assumption can effectively model the dropout evens of single-cell data [26, 37], which may also improve the performance of our model. Third, our model can be extended to incorporate other types of functional genomics data such as chromatin accessibility. For example, in the literature, the method of coupled nonnegative matrix factorizations performs clustering by the integrative analysis of scRNA-seq and single-cell ATAC-sequencing data [38]. Finally, the performance and efficiency of the model may be further improved by parameters inference using stochastic optimization and deep neural networks, which have been shown to be effective in statistical models for single-cell data analysis [39, 40].

## Conclusions

We have proposed a model-based algorithm, named VPAC, for accurate clustering of single-cell transcriptomic data through variational projection. Benefitting from the variational projection while constraining single-cell samples to follow a Gaussian mixture distribution in the latent space, VPAC is superior to existing methods in the clustering of datasets of discrete counts, normalized continuous data, various data dimensionality, different dataset size and different data sparsity. We have further demonstrated the ability of VPAC to detect genes with strong unique signatures of a specific cell type, which may shed light on the studies in system biology. Eventually, with the explosive growth of scRNA-seq data, we expect that such a statistical approach will provide us with superior performance and be widely applicable.

## Abbreviations

ARI: Adjusted rand index
CAVI: Coordinate ascent variational inference
ELBO: Evidence lower bound
FPKM: Fragments per kilobase per million mapped reads
NMI: Normalized mutual information
PBMCs: Peripheral blood mononuclear cells
PCA: Principal component analysis
PCC: Pearson correlation coefficient
PPCA: Probabilistic principal components analysis
scRNA-seq: Single-cell RNA-sequencing
SNN: Shared nearest neighbors
TPM: Transcripts per million mapped reads
t-SNE: t-Distributed Stochastic Neighbor Embedding
UMI: Unique molecular identifiers
